# The long-term impact of mild COVID-19 on cardiovascular disease and mortality in patients on hemodialysis: a post-Omicron era retrospective observational study

**DOI:** 10.1080/0886022X.2025.2512053

**Published:** 2025-06-02

**Authors:** Akiko Okamoto, Teppei Okamoto, Takaki Ichiyama, Takafumi Fukushima, Sosuke Omizo, Himawari Asanuma, Hiroyuki Sato, Hisashi Sakurai, Anna Yoneyama, Fumiya Yoneyama, Noritaka Ishii, Takuya Oishi, Ryuma Tanaka, Hikari Miura, Tomoko Hamaya, Hirotake Kodama, Naoki Fujita, Hiromi Murasawa, Hayato Yamamoto, Atsushi Imai, Hisao Saitoh, Shingo Hatakeyama, Chikara Ohyama, Tadashi Suzuki

**Affiliations:** a Department of Urology, Oyokyo Kidney Research Institute Hirosaki Hospital, Hirosaki, Japan; b Department of Urology, Hirosaki University School of Medicine, Hirosaki, Japan

**Keywords:** COVID-19, hemodialysis, cardiovascular disease, mild infection, Omicron variant

## Abstract

**Background:**

The long-term cardiovascular (CVD) and mortality risk associated with mild COVID-19 infections in patients on hemodialysis (HD) during the post-Omicron era remains unclear. This study evaluates clinical outcomes in patients on HD following Omicron infection.

**Methods:**

This retrospective observational study included 462 patients from a single center. All mild COVID-19 cases occurred after January 2022. The first analysis compared patients with prior mild COVID-19 (COVID-19 history [+], *n* = 63) to those never infected including the observational period (COVID-19 infection [−], *n* = 286). The second analysis included 392 patients without prior infection, comparing those who acquired mild COVID-19 during follow-up (COVID-19 infection [+]) with those in the COVID-19 infection [−] group. The primary outcome was CVD events. Multivariate analyses assessed COVID-19 infections’s impact on clinical outcomes.

**Results:**

After 761 days, 100 CVD events including 51 cardiovascular deaths and 58 non-CVD deaths occurred. The CVD risk did not significantly differ between the COVID-19 history (+) and infection (-) groups (Hazard ration [HR]: 0.50, 95% confidence interval [CI]: 0.25–1.01, *p* = .054) as well as non-CVD mortality. In the second analysis, patients with newly acquired COVID-19 did not exhibit a significantly increased risk of CVD (HR: 0.55, 95% CI: 0.25–1.19, *p* = .132) or non-CVD mortality.

**Conclusion:**

Mild COVID-19 infections does not significantly increase long-term CVD and mortality risk in patients on HD in the post-Omicron era.

## Introduction

COVID-19 infection can lead to a higher risk of death in people with chronic kidney disease (CKD) including patients on hemodialysis (HD) [1]. COVID-19 infection has been associated with systemic inflammation, endothelial dysfunction, and thrombotic events, leading to an increased risk of cardiovascular disease (CVD) in the general population [2,3]. Several studies have reported that individuals recovering from COVID-19 exhibit a higher incidence of myocardial infarction, stroke, and thromboembolic events, with these effects persisting for up to some years post-infection [4–6]. A large-scale cohort study indicated that post-acute sequelae of COVID-19 infection include a significantly elevated risk of CVD, particularly in those with severe infections requiring hospitalization including patients on hemodialysis (HD) [7–10]. Since late 2021, the COVID-19 pandemic landscape has dramatically shifted with the emergence of the Omicron variant, which is characterized by lower hospitalization and mortality rates. Furthermore, widespread vaccination campaigns and the introduction of effective antiviral therapies have significantly altered the clinical course of COVID-19 [11,12]. A recent report suggested that Omicron infections for mild cases do not lead to increased CVD events [12]. However, the clinical course of Omicron variant infections remains less understood, particularly in patients on HD.

Patients on HD already experience a high baseline risk of CVD due to factors such as vascular calcification, malnutrition, chronic inflammation, and a prothrombotic state [13–16]. Some reports have suggested that dialysis patients may experience attenuated inflammatory responses to infections due to chronic immune dysfunction [17,18]. Given these factors and findings, patients on HD might exhibit a different response to COVID-19 infection from the general population, determining whether even mild COVID-19 infections alter CVD risk in HD patients is of critical importance. This study aims to evaluate the long-term cardiovascular risk in patients on HD with mild COVID-19 infections and compare them to non-infected patients on HD.

## Materials and methods

### Subjects

This retrospective study utilized a database from a separate prospective cohort study. This study included 464 Patients on HD from Oyokyo Kidney Research Institute Hirosaki Hospital. Molnupiravir was approved in Japan in December 2021 and subsequently became widely used in patients on HD. Given the high risk of severe disease in this population, all patients on HD at risk of severe COVID-19 were prescribed the medication as part of their treatment regimen. This study was performed in accordance with the ethical standards of the Declaration of Helsinki and approved by the ethics review board of Hirosaki University School of Medicine (authorization number: 2022-004-1). We have already obtained written informed consent from all participants for the separate prospective cohort study. In this retrospective study, we followed an opt-out consent approach in accordance with ethical guidelines. All data used in this study were anonymized and stripped of any personally identifiable information to ensure strict patient confidentiality in accordance with applicable regulations.

### The classification of COVID-19 severity

In this study, we classified COVID-19 severity based on the World Health Organization criteria [19]. Mild COVID-19 was defined as cases with symptoms such as fever, cough, sore throat, headache, muscle pain, or loss of taste/smell, without pneumonia or hypoxia (SpO_2_ ≥94% on room air). Patients in this category did not exhibit signs of respiratory distress. Moderate COVID-19 was defined as cases presenting with clinical signs of pneumonia, such as fever, cough, and dyspnea, but without severe pneumonia. These patients maintained an oxygen saturation level (SpO_2_) of ≥90% on room air and did not require oxygen therapy. Severe and critical COVID-19 cases, which include patients with severe pneumonia (SpO_2_ <90% on room air), respiratory failure, septic shock, or multi-organ dysfunction requiring intensive care unit admission or mechanical ventilation. Severe cases were not observed in our cohort. As almost of all COVID-19 cases in our study were classified as mild, our analysis focused on comparing cases with mild COVID-19 with non-infected patients to evaluate their long-term CVD risk and all-cause mortality. We investigated the impact of moderate COVID-19 infection on clinical outcomes in the additional analysis.

### Patient classification and outcome assessment

In this study, patients were stratified into three groups based on their COVID-19 infection history. Our cohort included patients with mild COVID-19 who were infected from January 2022 onward, when Omicron variant was the predominant variant.

We conducted two main analyses: Long-term effects of past infection: A comparison of patients with a confirmed history of COVID-19 at baseline (COVID-19 history [+]) and those who remained uninfected throughout the entire study period (COVID-19 infection [−]). Short-term effects of acute infection: A subset analysis of patients without prior infection, comparing those who newly contracted COVID-19 during the observation period (COVID-19 infection [+]) with those who remained uninfected (COVID-19 infection [−]). Although COVID-19 vaccination was strongly encouraged in our institution, not all patients received three or more doses. We documented the number of COVID-19 booster vaccinations each patient had received at the beginning of the observation period. The follow-up period extended from December 2022 to February 2025 (761 days, 26 months). Additionally, we separately assessed the prognosis of patients with moderate COVID-19, as no severe cases were observed in our cohort. The primary outcome was the occurrence of cardiovascular disease (CVD) events. Cardiovascular (CVD) events were defined as clinically significant cardiovascular conditions associated with morbidity or mortality. Specifically, CVD events included acute coronary syndrome, such as myocardial infarction, sudden cardiac death, heart failure requiring hospitalization or resulting in death, clinically significant arrhythmias requiring pacemaker implantation or urgent treatment, stroke, severe valvular heart disease requiring interventional procedures or resulting in death, and symptomatic peripheral arterial disease. A history of CVD was defined as having at least one of these conditions at baseline. Clinical and demographic data, dialysis duration, comorbidities, and medication use were collected from medical records.

### Statistical methods

Demographic and laboratory parameters were summarized by median with interquartile range (IQR) and percentages for categorical variables. The significance of differences between the two groups was analyzed using the chi-squared test (categorical values), Student’s t-test (normally distributed data), and the Mann-Whitney test (non-normally distributed variables).

It is important to account for the presence of competing risks, particularly in the HD population, where non-CVD death can preclude the occurrence of cardiovascular CVD events. Accordingly, both CVD events and non-CVD deaths were analyzed using Fine–Gray subdistribution hazard models, treating the other cause of death as a competing risk in each case. Gray’s test was used to compare cumulative incidence functions between groups.

Furthermore, COVID-19 infection during the observation period cannot be used as a baseline covariate, but as a time-dependent covariate. To incorporate a time-dependent covariate, we used time-dependent covariate-included multivariable analyses in the second analysis. In all statistical tests, p-values <0.05 were considered statistically significant. In the analyses of CVD events, hazard ratios (HR) with 95% confidence intervals (CIs) were calculated after controlling for potential confounders, including age, sex, nutritional status (Geriatric Nutritional Risk Index; GNRI), diabetes mellitus (DM), history of CVD, calcium-phosphorous product (CaP product), C-reactive protein (CRP), past smoking history, the number of COVID-19 booster vaccinations, and HD vintage. In the analyses of non-CVD deaths, HR with 95% CIs were calculated after controlling for potential confounders, including age, sex, GNRI, DM, history of malignancy, and HD vintage. All analyses were performed using GraphPad Prism 5.03 (GraphPad Software, San Diego, CA, USA) and EzR (R commander version 1.6–3).

## Results

Of 464 patients, 2 were excluded due to a lack of the date of COVID-19 infection. Ultimately, 462 patients (296 males and 166 females) were evaluated. Among them, 68 patients had a history of COVID-19 infection before the observational period, with 5 cases classified as moderate and no cases classified as severe. In overall, during the 761-day observation period, 100 CVD events occurred, including 51 cardiovascular deaths. In addition, 58 non-CVD deaths were recorded.

**Figure 1.**
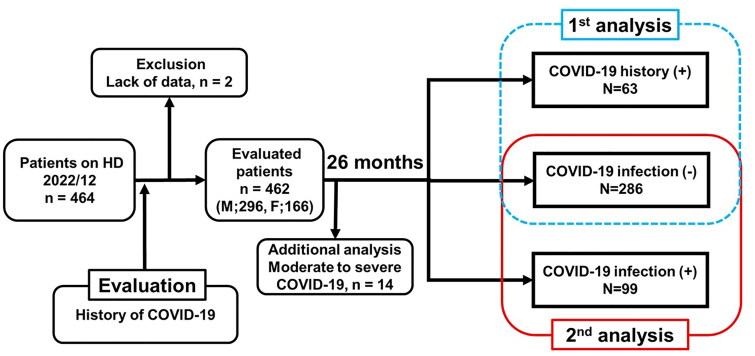
Patients’ classification and study design. Flowchart depicting patient classification. Patients on hemodialysis (HD) were categorized into three groups: (1) those with a prior history of COVID-19 (COVID-19 history [+]), (2) those who remained uninfected throughout the study period (COVID-19 infection [−]), and (3) those who newly acquired COVID-19 during follow-up. Two main analyses assessed the long-term effects of prior infection and the short-term impact of new infections. Moderate cases were analyzed separately; no severe cases were observed.

**Figure 2.**
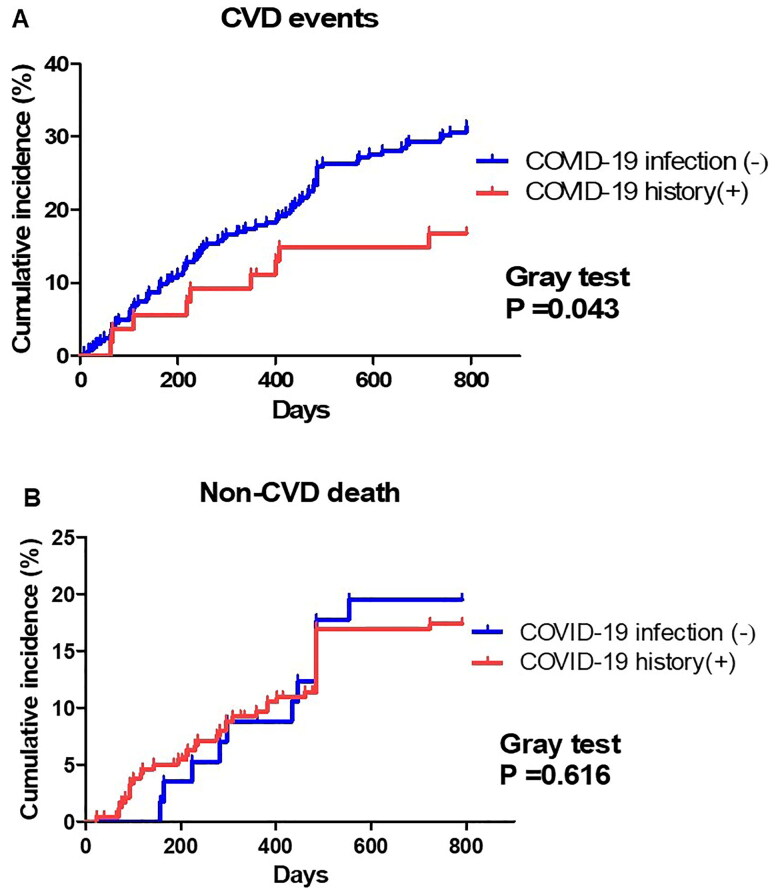
The cumulative incidence of CVD events and survival curve of all-cause of mortality in the first analysis. (A) Cumulative incidence curves comparing CVD events between the COVID-19 history (+) and COVID-19 infection (−) groups. CVD incidence was significantly lower in the COVID-19 history (+) group (*p* = .043). (B) Cumulative incidence curves comparing non-CVD death between the two groups (*p* = .616).

In the first analysis, we compared patients with a history of mild COVID-19 infection (COVID-19 history [+] group, *n* = 63) to those who had never been infected throughout the study period (COVID-19 infection [−] group, *n* = 286) (Figure 1). Table 1 shows the characteristics of the first analysis, with a significant difference observed in the number of the booster vaccinations between the groups. In total, 84 CVD events and 47 non-CVD deaths occurred in the cohort. The cumulative incidence of CVD events in the COVID-19 history (+) group was significantly lower than that in the COVID-19 infection (−) group (*p* = .043, Figure 2A). However, non-CVD deaths did not differ significantly between the two groups (*p* = .616, Figure 2B). Fine–Gray proportional hazards regression analysis, accounting for non-CVD deaths as a competing risk, showed that neither a history of COVID-19 infection (HR: 0.50, 95% CI: 0.25–1.01, *p* = .054, Table 2) nor the number of booster vaccinations had a significant impact on the incidence of CVD events. Fine-Gray proportional hazards regression analysis, accounting for non-CVD deaths as a competing risk, showed no significant impact of COVID-19 history on the CVD events, as well as the numbers of booster vaccinations. Fine-Gray proportional hazards regression analysis confirmed that a history of COVID-19 infection was not an independent risk factor for non-CVD deaths (HR: 1.67, 95% CI: 0.90–3.10, *p* = .100, Table 2).

**Table 1.** The patients’ characteristics of the first analysis.

	COVID-19 infection (−)	COVID-19 history (+)	*p* value
N	286	63	
Age^b^ (years) (median, IQR)	71 (61–77)	70 (60–79)	.963
Male sex^a^	178 (62%)	43 (68%)	.390
History of CVD^a^	150 (52%)	30 (48%)	.491
HD vintage^a^ (month, median, IQR)	5.2 (2.5–10.3)	5.1 (2.7–9.2)	.969
Past smoking history	173 (61%)	40 (64%)	.776
Cause of end-stage renal disease (DM)^a^	137 (48%)	27 (44%)	.579
Presence of DM^a^	154 (54%)	32 (49%)	.577
Hypertension^a^	104 (64%)	45 (65%)	.899
History of malignancy^a^	58 (20%)	8 (13%)	.213
Body mass index^b^ (median, IQR)	21.7 (19.5–24.8)	21.1 (19.0–23.3)	.247
Serum albumin level^b^ (median, IQR)	3.5 (3.3–3.7)	3.5 (3.4–3.7)	.681
GNRI^b^ (median, IQR)	92.3 (86.4–96.3)	92.6 (87.8–94.9)	.959
CaP product^b^ (median, IQR)	50.9 (40.5–61.6)	50.4 (40.3–56.5)	.439
Hemoglobin levels^b^ (median, IQR)	11.4 (10.7–12.2)	11.5 (10.7–12.0)	.841
CRP^b^ (median, IQR)	0.15 (0.05–0.51)	0.12 (0.04–0.34)	.361
The number of booster vaccinations at the beginning of the observation period.			.018
0	28 (10%)	2 (3.2%)	
1	5 (1.7%)	2 (3.2%)	
2	7 (2.4%)	6 (9.5%)	
3	16 (5.6%)	6 (9.5%)	
4	230 (80%)	47 (75%)	
Non-CVD deaths^a^	41 (14%)	6 (10%)	.558
Total CVD events^a^	75 (26%)	9 (14%)	.119
Peripheral arterial diseases^a^	10 (13%)	1 (11%)	
Coronary arterial diseases^a^	8 (11%)	1 (11%)	
Heart failure^a^	27 (36%)	3 (33%)	
Sudden cardiac death^a^	7 (9.3%)	2 (22.2%)	
Stroke^a^	8 (11%)	1 (11%)	
Arrythmia^a^	9 (12%)	0 (0%)	
Valvular heart disease^a^	6 (8.0%)	1 (11%)	
			

IQR: Interquartile range; CVD: cardiovascular, HD: hemodialysis, DM: diabetes mellitus, GNRI: Geriatric Nutritional Risk Index, CRP: C-reactive protein, CaP product: calcium-phosphorous product.

^a^Chi-squared test, ^b^Mann-Whitney test.

**Table 2.** Multivariate analyses of 1st analysis (COVID-19 history [+] vs. COVID-19 infection [−]).

Fine Gray proportional hazard analysis for CVD events (1st analysis)				
Variables	Factor	Hazard ratio	95% CI	*p* value
Age (years)	Continuous	1.03	1.01–1.05	.0005
The number of booster vaccinations	Continuous	0.96	0.81–1.14	.640
COVID-19 history	presence	0.50	0.25–1.01	.054
Fine Gray proportional hazard analysis for non-CVD deaths (1st analysis)				
Variables	Factor	Hazard ratio	95% CI	*p* value
Age (years)	Continuous	1.06	1.06–1.09	<.001
GNRI	Continuous	0.89	0.87–0.92	<.001
COVID-19 history	presence	1.67	0.90–3.10	.100

The model for CVD events were adjusted by age, sex, Geriatric Nutritional Risk Index (GNRI), diabetes mellitus (DM), history of CVD, calcium-phosphorous product (CaP product), C-reactive protein (CRP), past smoking history, and hemodialysis (HD) vintage.

The model for non-CVD deaths were adjusted by age, sex, GNRI, DM, history of malignancy, and hemodialysis (HD) vintage.

During the 761-day follow-up period, 108 patients acquired COVID-19, including 99 mild cases (COVID-19 infection [+] group, Figure 1) and 9 moderate cases. No patients developed severe COVID-19 or died from COVID-19 infection during the observation period. Therefore, we compared 99 patients in the COVID-19 infection (+) group to 286 patients in the COVID-19 infection (-) group in the second analysis. In total, 91 CVD events and 45 non-CVD deaths occurred in the cohort. Time-dependent covariate-adjusted Fine–Gray proportional hazards regression analysis, accounting for non-CVD deaths as a competing risk, demonstrated that neither COVID-19 infection (HR: 0.55, 95% CI: 0.26–1.20, *p* = .130; Table 3) nor the number of booster vaccinations significantly affected the incidence of CVD events. Furthermore, the same model confirmed that COVID-19 infection was not independently associated with non-CVD death (HR: 0.99, 95% CI: 0.43–2.29, *p* = .985; Table 3). Table 4 shows summary of cardiovascular events and deaths among COVID-19-positive patients in Analysis 1 and 2. No events occurred within 1 year of infection in either group, suggesting minimal acute or subacute impact of COVID-19 on outcomes.

**Table 3.** Time-dependent covariate-included multivariate analyses of 2nd analysis (COVID-19 infection [+] vs. COVID-19 infection [−]).

Fine Gray proportional hazard analysis for CVD events (2nd analysis)				
Variables	Factor	Hazard ratio	95% CI	*p* value
Age (years)	Continuous	1.02	0.99–1.05	.031
History of CVD	Presence	1.79	1.14–2.81	.011
The number of booster vaccinations	Continuous	0.98	0.83–1.18	.900
COVID-19 infection	Presence	0.55	0.25–1.19	.132
Fine Gray proportional hazard analysis for non-CVD deaths (2nd analysis)				
Variables	Factor	Hazard ratio	95% CI	*p* value
Age (years)	Continuous	1.06	1.03–1.11	.003
GNRI	Continuous	0.90	0.87–0.93	<.001
COVID-19 infection	Presence	0.99	0.43–2.23	.985

The model for CVD events were adjusted by age, sex, Geriatric Nutritional Risk Index (GNRI), diabetes mellitus (DM), history of CVD, calcium-phosphorous product (CaP product), C-reactive protein (CRP), past smoking history, and hemodialysis (HD) vintage.

The model for non-CVD deaths were adjusted by age, sex, GNRI, DM, history of malignancy, and hemodialysis (HD) vintage.

**Table 4.** Summary of cardiovascular events and deaths among COVID-19–positive patients (1st and 2nd analysis).

Analysis	COVID-19 Group	Total Patients	CVD Events (New-Onset)	Non-CVD Deaths	Events Within 1 Year	Mean Time to CVD (days)	Mean Time to Death (days)
1st analysis	Prior COVID-19 infection before observation	63	9 (7 after infection)	9	0	283	305
2nd analysis	COVID-19 acquired during observation	99	16 (7 after infection)	6	0	284	211

CVD: cardiovascular disease. New-onset CVD refers to events occurring after the COVID-19 infection date. Events within 1 year = number of CVD or death events occurring within 365 days of COVID-19 infection.

## Discussion

This study demonstrates that both a history of mild COVID-19 infection and newly acquired COVID-19 during the Omicron era were not independent risk factors for CVD events or all-cause mortality in patients on HD. Our results suggest that mild COVID-19 infection does not significantly alter CVD risk or mortality in patients on HD. To our knowledge, this is the first study to comprehensively assess the long-term cardiovascular impact of Omicron-variant COVID-19 in patients on HD.

Several prior studies have established an association between COVID-19 infection and increased cardiovascular risks and all-cause mortality in patients with CKD [1]. Carriazo et al. reported a 35.7% one-year mortality rate, with deaths occurring both during hospitalization and in the months following infection [10]. Lambourg et al. conducted a large-scale multi-regional study and found that COVID-19 infection doubled the risk of cardiovascular death within 30 days of infection and remained significantly elevated long-term [8]. Similarly, Del Vecchio et al. suggested that COVID-19 increases thrombotic and myocardial complications in patients with CKD through mechanisms including endothelial damage, persistent inflammation, and hypercoagulability [20]. Similarly, a study by Rao et al. found that while kidney disease was associated with severe COVID-19 outcomes [1]. However, since these studies focused on data from the early period of the COVID-19 pandemic, their findings may not be directly applicable to the Omicron era and the near future.

Several recent studies have highlighted the long-term cardiovascular, cerebrovascular, and thrombotic complications of COVID-19 post the Omicron era [11,12,21]. An earlier study during the Delta wave suggested that COVID-19 infection substantially increased cardiovascular and cerebrovascular risks, but vaccination significantly mitigated these risks [11]. Conversely, a retrospective cohort study in Singapore investigated post-Omicron cardiovascular risks and found that while new-onset cardiovascular complications were not significantly increased, there was a slight elevation in the risk of arrhythmias [12]. Our study also demonstrates that both a history of mild COVID-19 infection and newly acquired COVID-19 during the Omicron era were not independent risk factors for CVD events or non-CVD deaths even in patients on HD. These findings may be attributed to several factors beyond the characteristics of the variants. In the Omicron era, over 80% of patients in our study had received three or more doses of the COVID-19 vaccine, and all patients who contracted COVID-19 received appropriate antiviral therapy. Moreover, some patients received additional booster doses during the observation period. Although we did not observe a statistically significant association between the number of vaccinations and CVD events, these preventive measures may have played a key protective role in reducing the risk of CVD, even in these patients [5,6].

Given that patients on HD are more vulnerable to CVD than the general population, the findings of this study were somewhat unexpected. Chawki et al. followed patients on HD who had recovered from COVID-19 before the Omicron era and found that 9.8% experienced CVD events within six months post-infection [9], which a rate that is not significantly higher than expected in this population [22]. Our sensitivity analyses showed that neither CVD events nor non-CVD deaths occurred within one year of infection, suggesting that mild COVID-19 during the post Omicron era did not have a substantial acute or early subacute impact in our cohort. Among 14 patients with moderate COVID-19 in our study, 7 died from CVD and 3 died from non-CVD causes, resulting in a mortality rate of 71%. The median time from infection to death was 508 d (Figure S1). This finding underscore that moderate COVID-19 can still have a serious long-term impact, even after the emergence of the Omicron variant.

This study has several strengths. First, we distinguished between patients with a prior history of COVID-19 and those who newly acquired COVID-19 during the observation period. This approach allowed us to evaluate both preexisting and time-dependent effects of infection, which may strengthen the validity of our conclusions. However, this study also has several limitations. First, it was a single-center retrospective study, which may limit the generalizability of the findings. Multi-center studies with larger and more diverse populations are needed to confirm our results. Second, although we adjusted for key clinical confounders in our multivariable models, residual confounding cannot be completely ruled out. Third, the number of COVID-19–positive cases, particularly those with moderate disease (*n* = 14), was small, limiting the statistical power to detect significant differences in this subgroup. Fourth, no patients with severe COVID-19 were included, as such cases were either rare during the Omicron era or treated at external institutions. Therefore, our findings apply primarily to mild or moderate cases. Fifth, the long interval between moderate infection and death (median: 508 d) in some patients raises uncertainty about the causal relationship between infection and mortality. Due to the small sample size, we were unable to perform subgroup-specific competing-risk analyses to clarify this relationship.

Despite these limitations, our study provides novel clinical evidence suggesting that both prior and newly acquired mild COVID-19 infections during the post-Omicron era do not significantly increase the risk of CVD events or mortality in patients in HD. In conclusion, this study showed that COVID-19 infection does not significantly impact the long-term risk of CVD or mortality in patients on HD. Considering the advancements in therapy and vaccination against COVID-19, as well as the decline in severe cases and mortality rates in recent years, our findings might help predict future trends in patients on HD.　This research may encourage researchers to clarify whether Omicron-era infections in patients on HD carry similar cardiovascular risks as reported in the general population in multi-center, long-term studies.

## Supplementary Material

Supplemental Material

## Data Availability

The data that support the findings of this study are available from the corresponding author upon reasonable request.
