# Supplementary figures and images for: The long-term impact of mild COVID-19 on cardiovascular disease and mortality in patients on hemodialysis: a post-Omicron era retrospective observational study
Source: Ren Fail. 2025 Jun 2;47(1):2512053. doi: 10.1080/0886022X.2025.2512053 (PMC12135085; doi:10.1080/0886022X.2025.2512053)

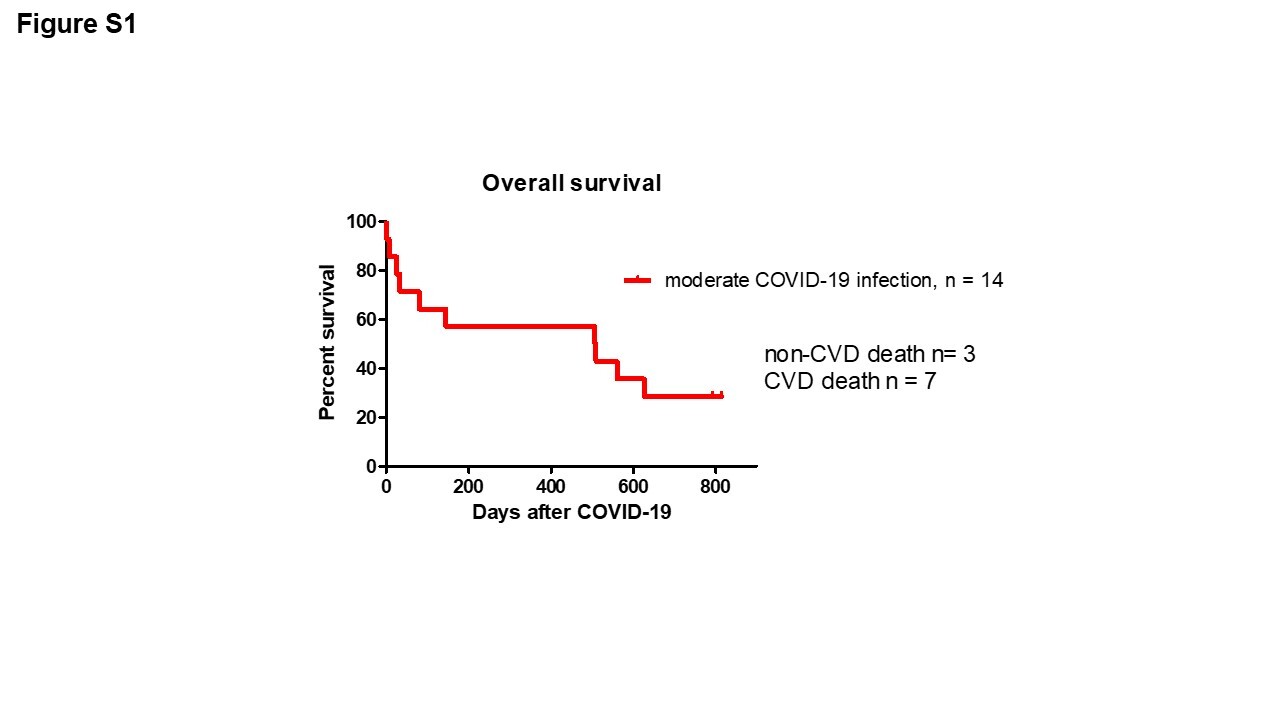

Supplement: Supplemental Material [file IRNF_A_2512053_SM8971.jpg]
